# A New Severity Index for Placental Abruption Predicts Recurrence Risk in Future Pregnancies

**DOI:** 10.3390/jcm15135308

**Published:** 2026-07-07

**Authors:** Lior Kerber, Shai Rippel, Ori Cohen, Lior Rokach, Efrat Spiegel, Taeer Avnon, Offer Erez

**Affiliations:** 1 Faculty of Health Sciences, Joyce & Irving Goldman Medical School, Ben-Gurion University of the Negev, Beer Sheva 84105, Israel; kerber@post.bgu.ac.il (L.K.); taeer.avnon@gmail.com (T.A.); 2 Department of Information Systems and Software Engineering, Ben-Gurion University of the Negev, Beer Sheva 84105, Israel; shairip@post.bgu.ac.il (S.R.); orioric@gmail.com (O.C.); liorrk@bgu.ac.il (L.R.); 3 Department of Obstetrics and Gynecology, Soroka University Medical Center, Ben-Gurion University of the Negev, Beer Sheva 84105, Israel; spiegele@post.bgu.ac.il; 4 Department of Obstetrics and Gynecology, Hutzel Women’s Hospital, School of Medicine, Wayne State University, Detroit, MI 48202, USA; 5 Obstetric High Risk Pregnancies Clinics, Division of Obstetrics and Gynecology, Soroka University Medical Center, Beer Sheva 84101, Israel

**Keywords:** preeclampsia, DIC, maternal death, hypoxic ischemic encephalopathy, perinatal death, blood transfusion, preterm birth, recurrent abruption

## Abstract

**Background:** Placental abruption (PA) is an unpredictable obstetric complication associated with substantial maternal and fetal/neonatal morbidity and mortality in the index and subsequent pregnancies. The previous PA severity classification categorizes more than two-thirds of cases as severe, which limits its clinical utility despite its association with adverse maternal outcomes. **Objective:** We aimed to develop a novel, clinically applicable PA severity classification and to determine its ability to predict its recurrence in subsequent pregnancies. **Study design:** This retrospective, population-based cohort study included medical records of all women who delivered at our medical center from 2001 to 2021. Pregnancies complicated by chromosomal abnormalities or major congenital anomalies were excluded. The novel PA severity classification included maternal (transfusion of ≥4 units of red blood cells, admission to an intensive care unit, DIC, hysterectomy, or maternal death) and neonatal (birth asphyxia, hypoxic–ischemic encephalopathy, umbilical cord blood acidosis defined as pH < 7.0, neonatal intensive care unit admission at ≥35 weeks gestation, or perinatal death) complications. Maternal characteristics, clinical features, and perinatal outcomes were compared between the novel and the existing PA severity classifications. A multivariate regression model was used to determine the association between PA severity and the risk of its recurrent in subsequent pregnancies. **Results:** Our cohort included 298,281 deliveries; of them, 1567 (0.5%) had PA. The rate of severe PA was lower in the novel vs. the existing classification (*p* < 0.001). Within the novel classification, severe PA was associated with higher rates of mild and severe preeclampsia and lower mean fibrinogen concentrations and platelet counts compared with mild abruption (all *p* < 0.001). Severe PA was independently associated with an increased risk of recurrence in subsequent pregnancies (OR 1.82, 95% CI 1.05–3.14). **Conclusions:** We propose a novel, clinically relevant PA severity classification that accurately identifies severe disease providing prognostic information regarding the risk of PA recurrence in future pregnancies.

## 1. Introduction

Placental abruption is a leading cause of maternal, fetal, and neonatal morbidity and mortality affecting 7–12/1000 pregnancies in the United States [1,2,3]. Risk factors for placental abruption include prior abruption, high parity, advanced maternal age, smoking, previous cesarean section, gestational hypertension, preeclampsia, trauma during pregnancy, preterm premature rupture of membranes (PPROM), polyhydramnios, cocaine and drug use, and chronic hypertension [3,4,5,6,7,8]. The diagnosis of placental abruption is primarily clinical, based on findings such as vaginal bleeding, uterine contractions, abdominal pain, and abnormal fetal heart rate tracings [1,9,10]. Other diagnostic parameters have limited usefulness [11]. Laboratory hemostatic parameters are not currently part of the diagnostic criteria for placental abruption but are included in the clinical assessment of the severity of maternal hemorrhage and hemodynamic stability [5]. Maternal complications include massive blood transfusion, disseminated intravascular coagulation (DIC), Intensive Care Unit (ICU) admission, end-organ damage, and death [3,5]. Perinatal complications include fetal death, complications of prematurity, birth asphyxia, and perinatal death; long-term complications include neurodevelopmental impairment and cerebral palsy [5,12,13]. Women who had placental abruption have a recurrence rate of 3–17%, reaching 25% after two previous episodes [14]. However, the risk factors for recurrent placental abruption in subsequent pregnancies remain unclear [14,15].

Maternal and fetal/neonatal outcomes are affected by the severity of the abruption [1,5,13]. Previous classification systems for placental abruption severity had a poor correlation with adverse outcomes [13,16,17,18]. The placental abruption severity classification that was recently introduced by Ananth et al. categorizes two-thirds of cases as severe, limiting its clinical implementation [13]. Therefore, there is a need for a placental abruption severity classification index that is easy to use, uses the maternal and perinatal complications that define the subgroup with the highest need for monitoring and intervention, and is informative of its recurrence. Therefore, we developed in a population-based cohort study a novel, easy-to-use classification index of placental abruption severity.

## 2. Materials and Methods

This retrospective, population-based cohort study included medical records of all women who delivered at our medical center from 2001 to 2021. Pregnancies complicated by chromosomal abnormalities or major congenital anomalies were excluded.

### 2.1. Novel Classification of Placental Abruption Severity

Placental abruption was classified as severe when at least one of the following complications was present. Maternal complications included (1) transfusion of ≥4 units of red blood cells; (2) admission to the ICU; (3) DIC; (4) hysterectomy; and (5) maternal death [19]. Perinatal complications included (1) birth asphyxia, hypoxic-ischemic encephalopathy; (2) severe cord blood acidosis (pH < 7.0); (3) admission to the neonatal intensive care unit (NICU) ≥ 35 weeks of gestation; and (4) perinatal death [20,21,22]. The new severity criteria were compared to the placental abruption severity classification as described by Ananth et al.—placental abruption accompanied by ≥1 of the following: DIC, hypovolemic shock, blood transfusion, hysterectomy, renal failure, maternal death, non-reassuring fetal status, fetal growth restriction, fetal death, neonatal death, preterm delivery, or small-for-gestational-age birth [13].

### 2.2. Data Collection

This study included all women who delivered between 2001 and 2021 at our medical center. Clinical data were collected from maternal Electronic Medical Records (EMR). All pregnancies and deliveries occurred at our medical center, a large tertiary hospital in the southern region of Israel where almost all deliveries of the region take place. Exclusion criteria were pregnancies with diagnosed congenital malformations or chromosomal abnormalities. The International Classification of Disease, 9th version (ICD-9) was used to code all diagnoses and procedures in the database. The diagnosis of placental abruption was obtained from maternal EMR using the ICD-9 code 64121 (‘Premature separation of placenta, delivered, with or without mention of antepartum condition’). The diagnosis was based on clinical suspicion and was confirmed by the attending physician, typically supported by postpartum findings such as retroplacental hematoma or placental separation. In addition, trained data personnel systematically review the medical records and ICD coding to ensure standardized documentation according to institutional diagnostic criteria. The study population was divided into two groups: women with (study) and without (comparison) placental abruption. Cases with placental abruption were further classified as mild and severe abruption according to the Ananth et al. classification and our classifications. The study was approved by the Institutional Review Board of Soroka University Medical Center (request number SOR-0113-22, approval date—3 January 2023).

### 2.3. Statistical Analysis

Analysis was performed using Python 4.7 (“Scikit-learn” 0.21.2 and “Pandas” 2.0.3 libraries) and R 4.3 (“gtsummary” library). Differences in demographics, pregnancy complications, delivery and perinatal characteristics, severity criteria, and laboratory studies were compared between deliveries with severe and mild placental abruption according to Ananth et al. and the new classification systems, along with deliveries without abruption (the comparison group). Continuous normally distributed variables are presented as the mean ± standard deviation (SD). Discrete and ordinal variables are presented as the median (and interquartile range) and categorical variables as numbers (%). One-Way ANOVA or Kruskal–Wallis were performed for the analysis of continuous variables with a post hoc T-test or Mann–Whitney rank-sum test with Bonferroni corrections, Wilcoxon test for ordinal variables and Pearson’s chi-square test or Fisher’s exact for categorical variables. A two-sided *p* value < 0.05 was considered statistically significant.

Logistic regression analysis was performed to evaluate the association between abruption in future pregnancies and potential predictors, including severe abruption in current pregnancy, parity, preeclampsia, and chronic hypertension. Only women with subsequent pregnancies were included. Odds ratios (ORs) with 95% confidence intervals (CIs) were calculated for each variable. Model coefficients were exponentiated to derive ORs, and *p*-values were reported for significance testing.

## 3. Results

The study cohort included 298,281 deliveries of 302,693 neonates who met the inclusion criteria. The rate of placental abruption was 0.5% (1567/298,281); of them, 28.7% (449/1567) were classified as severe by us vs. 82.2% (1288/1567) by Ananth et classification (*p* < 0.001).

Table 1 describes the demographic characteristics and medical history of the study population. According to the novel classification, women with severe placental abruption had a significantly higher median gravidity and parity (*p* < 0.001 for both) and previous perinatal death (*p* = 0.03) than those with mild abruption. Women classified as having severe abruption had a higher rate of prior placental abruption than those in the comparison group.

Table 2 describes the pregnancy and delivery characteristics of patients with severe and mild placental abruption. In both classifications, the rate of severe preeclampsia was higher in women with severe abruption than in those with mild abruption. However, the rate of mild preeclampsia was higher in severe abruption cases than in mild abruption cases in the novel classification only. The rates of preterm PROM were lower in severe cases than in those with mild abruption according to the novel classification.

Table 3 describes the neonatal characteristics of the study groups. According to Ananth et al.’s classification, severe abruption was associated with a lower mean gestational age at delivery (33.5 ± 4.8 and 38.5 ± 2.2, respectively, *p* < 0.001) and birthweight (2018.9 ± 880 and 3081.6 ± 533, respectively, *p* <0.001) compared to mild abruption. According to the novel classification, the differences in mean gestational age at delivery and birthweight between mild and severe abruption did not reach statistical significance. The differences in mean birth weight between the two groups were also not significant. The rate of small for gestational age (SGA) was significantly higher in the severe abruption group according to both classifications.

Table 4 describes hemostatic laboratory parameters according to the study groups. In both classifications, patients with severe abruption had a lower mean hemoglobin concentration (*p* < 0.001), hematocrit (*p* < 0.001), and fibrinogen concentration (Ananth’s 502.3 mg/dL and 558.4 ± 107 mg/dL respectively, *p* < 0.001, novel 431.7 ± 170 mg/dL and 547.5 ± 125 mg/dL, respectively, *p* < 0.001). Hypofibrinogenemia < 100 mg/dL was also higher among patients with severe abruption than among those with mild abruption according to the novel classification (2.0% vs. 0%, respectively, *p* < 0.001). Platelet count was significantly lower in patients with severe abruption compared to those with mild abruption according to the novel classification only (209.8 ± 72 103/uL and 232.1 ± 64.6 103/uL, respectively, *p* < 0.001). The rates of thrombocytopenia (<100 × 10^3^/µL) were significantly higher among severe compared to mild abruption according to both classifications (1.9% vs. 0%, respectively, *p* = 0.4 for Ananth’ classification and 4.2% vs. 0.5%, respectively, *p* < 0.001 for the novel classification).

Table 5 describes the differences in the clinical characteristics used for the diagnosis of severe abruption by each classification method. The rates of any blood transfusions, transfusion of ≥four blood units, maternal ICU admission, NICU admission after 35 weeks of gestation, perinatal death, and DIC are higher in patients with severe abruption in both classifications. The novel classification showed a greater difference between severe and mild abruption in all these parameters. The rates of birth asphyxia, umbilical cord blood pH < 7, hysterectomy, diagnosis of hypovolemic shock, and renal failure are significantly higher in severe abruption according to the novel classification but not according Ananth’s classification. The rates of fetal growth restriction and preterm delivery were significantly higher among patients with severe abruption according to Ananth’s classification. Notably, the most prevalent features according to Ananth’s classification were preterm delivery in 66% (854 cases), and non-reassuring fetal status in 34% (442 cases), compared with our classification, where perinatal death occurred in 59% (264) and NICU admission > 35 weeks occurred in 25.4% (114 cases).

**Figure 1:**
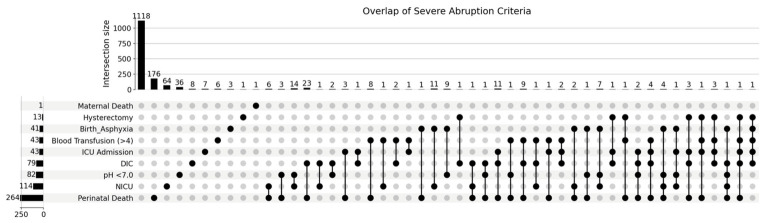
Uset plot for the interaction among the criteria for placental abruption severity.

The rates and interaction of placental abruption severity classification features are presented in Figure 1. Perinatal death is the most dominant feature and accounts for the diagnosis of 264 cases as severe; of them, 176 cases were isolated and did not overlap with other severity features.

The association between placental abruption severity and its recurrence in subsequent pregnancies after adjusting for confounding factors is presented in Table 6. Among 1567 women, 1503 had subsequent pregnancies with 57 (3.8%) women experienced recurrent abruption. According to the our classification recurrence risk was 5.5% (23/420) in severe abruption and 3.1% (34/1083) in mild abruption. In comparison, according to Ananth’s classification recurrence riskwas 4.1% (50/1236) in severe abruption and 2.6% (7/267) in mild abruption. Logistic regression was performed including severe abruption in index pregnancy, parity, preeclampsia, and chronic hypertension as dependent variables. Severe abruption in the index pregnancy, is an independent risk factor of recurrent abruption according to our classicfication only (OR 1.82, 95% CI 1.05–3.14; *p* = 0·03).

## 4. Discussion

### 4.1. Principle Findings of the Study

We developed a novel classification for placental abruption severity including nine maternal and perinatal parameters. Our method classified approximately 30% of cases as severe abruption and, compared to Ananth et al.’s [13] classification, demonstrated a stronger association with severe features of abruption including severe preeclampsia, as well as abnormal hemostatic status (i.e., low mean fibrinogen concentration and platelet count). Moreover, it has clinical implications for subsequent pregnancies as severe abruption is an independent risk factor for recurrent placental abruption.

### 4.2. Results in the Context of What Is Known

The diagnosis of placental abruption is clinical, and the contribution of bedside ultrasound examination antepartum is limited [9,10]. However, severe cases of placental abruption are associated with increased maternal, fetal, and neonatal morbidity and mortality. Therefore, there is a need for a pragmatic and clinically meaningful severity classification that can reliably differentiate between clinical degrees of placental abruption severity and facilitate standardized research across populations. Such a framework may also provide a foundation for the future development of clinically applicable risk stratification tools.

Prior attempts to classify the severity of placental abruption were not implemented into clinical practice. Indeed, Pritchard et al. proposed defining severe abruption as abruption complicated by stillbirth and identified external trauma, sudden uterine decompensation, maternal vascular disease, high parity, and previous abruption as risk factors for severe abruption [17], and others have used a scoring system ranging from zero to three based on maternal symptoms and fetal distress [17,18]. The restrictive nature of latter classification potentially leads to the oversight of severe perinatal outcomes since it did not include perinatal criteria other than fetal distress [13,16]. Ananth et al.’s classification was based on a retrospective cohort analysis of large-scale data. Severe abruption was defined according to the presence of maternal (DIC, hypovolemic shock, blood transfusion, hysterectomy, renal failure, or in-hospital death), fetal (nonreassuring fetal status, intrauterine growth restriction, or fetal death), or neonatal (neonatal death, preterm delivery, or small for gestational age) complications [13]. According to this classification, severe abruption was associated with a higher risk for maternal cerebrovascular, cardiac, or pulmonary morbidity. However, due to the inclusive nature of this classification, about two-thirds of cases were categorized as ‘severe abruption,’ limiting its implementation in clinical settings [13]. One possible explanation is the inclusion of parameters that are not specific to abruption severity, such as nonreassuring fetal status, which is frequently documented but has a low positive predictive value for fetal asphyxia [23], or, on the other hand, preterm birth, which, although it may be associated with severe neonatal outcomes, is also part of the natural process of placental abruption. Moreover, when this classification was applied to our cohort, cases of placental abruption accompanied by neonatal birth asphyxia, umbilical cord blood pH < 7, NICU admission, or maternal ICU admission were classified as mild rather than severe abruption, suggesting that this classification may be less sensitive to the neonatal impact of placental abruption and its implications on maternal status (Table 5). It is important to note that our classification was not evaluated for the prediction of morbidity profiles, unlike Ananth’s classification, as it includes maternal ICU admission, which encompasses all severe maternal morbidities.

Our classification of placental abruption severity brings an original approach. We included parameters of maternal and perinatal complications that were previously described to be associated with severe abruption and adverse obstetric outcomes [19,20,21,24,25,26]. Maternal criteria included obstetrical hemorrhage and coagulopathy requiring the infusion of ≥4 blood units and ICU admissions. These demonstrated high specificity and sensitivity in identifying women with severe obstetrical morbidity [19,24,25,26]. We emphasize the parameters of the maternal hemostatic system as they are directly associated with maternal morbidity and mortality and play a key role in the management of placental abruption [1,5]. Indeed, DIC is one of the life-threatening outcomes of abruption. Previous reports suggested that placental abruption is a leading cause of DIC in pregnancy, especially when associated with fetal death [27,28,29,30]. The pathophysiology of DIC is associated with increased thrombin generation and the degradation of coagulation factors and anticoagulation proteins, followed by their impaired synthesis, leading to uncontrolled bleeding [27,31]. Maternal shock and its complications play a significant role in the management of placental abruption. The diagnosis and severity of DIC, a life-threatening complication of hemorrhagic shock, is based on laboratory hemostatic parameters including platelet count, prothrombin-time (PT), and fibrinogen, and a pregnancy-specific DIC scoring system assigned fibrinogen concentration as the most indicative blood test for DIC severity, followed by prothrombin-time elongation and platelet count [32]. In the context of abruption, the decrease in platelet counts and hypofibrinogenemia along with prolonged PT occurs faster than in other conditions and requires a greater amount of blood product transfusion compared to other conditions causing DIC [29,33]. Therefore, the presentation of DIC in the setting of abruption may indicate massive bleeding and the need for prompt intervention [27,31,32]. The rate of DIC among patients classified as having severe abruption by the novel classification was 18%, and only 6.3% according to Ananth et al.’s classification, suggesting that the novel classification is more sensitive to cases with severe abruption and adverse outcome.

Moreover, we included peripartum hysterectomy resulting from severe and uncontrolled obstetrical hemorrhage as a life-saving procedure and can serve as an indicator for severe maternal morbidity [1].

Placental abruption and preeclampsia are part of placental-mediated obstetrical syndromes that share common maternal and fetal vascular lesions of placental malperfusion [34,35,36,37]. Indeed, the rates of placental abruption are higher among patients with severe preeclampsia than in those with normotensive pregnancies [28,34,35]. We propose that the mechanism associating placental abruption and preeclampsia may mimic placental hemorrhagic stroke resulting from spasms of the uteroplacental vessel and subsequent rupture and bleeding. This is an acute event associated with massive maternal hemorrhage and, if severe enough, can lead to fetal distress, birth asphyxia, perinatal death, and maternal coagulopathy [38,39]. Preeclampsia, in both mild and severe forms, is highly associated with severe placental abruption according to the novel classification, and its rate among severe cases is higher than that observed based on Ananth et al.’s classification.

The perinatal criteria included in our classification relate mostly to birth asphyxia, which is linked to short- and long-term adverse perinatal outcomes [20]. Indeed, birth asphyxia is considered as the major cause of neonatal death and long-term morbidity associated with placental abruption [20]. Umbilical artery pH < 7.0 is an additional indicator of fetal hypoxic acidemia that accompanied 17% of abruption cases with asphyxia [21]. Placental abruption that resulted in birth asphyxia is associated with an increased risk of long-term neonatal morbidity such as cerebral palsy [21].

From a neonatal perspective, prematurity is a known complication of preterm placental abruption that affects its management [1], and the association between preterm PROM and placental abruption is well defined. Previous studies have demonstrated distinctions between term and preterm abruption, showing that acute inflammatory processes such as those related to preterm PROM are more prominent in preterm placental abruption [40]. Moreover, Ananth et al. recently presented a comprehensive approach for term placental abruption as a distinct type of abruption, which in many cases results from chronic vascular sequela such as chronic hypertension [5]. However, in a high proportion of the cases, preterm PROM may be the end-stage clinical presentation of subacute, chronic abruption [7,25]. Indeed, the rate of preterm PROM was associated with the milder form of abruption in our classification. Early gestational age at delivery is by itself a risk factor for perinatal morbidity and mortality. Indeed, Ananth’s classification considered preterm birth in the outcome data for the definition of severe abruption [13]. However, a previous study demonstrated that abruption increases the risk for perinatal mortality regardless of gestational age, overcoming the inherent risk of prematurity [41]. Therefore, in our novel classification, we included perinatal mortality as a parameter for the severity of abruption rather than gestational age at delivery [12,42]. Moreover, to address the contribution of prematurity to fetal/neonatal morbidity, we included only NICU admissions after 35 weeks of gestation as a marker for severity, since at earlier gestational ages, all neonates are admitted to the NICU in our medical center due to prematurity, regardless of the severity of abruption. Previous reports suggested inter-hospital variation in NICU admission rates among infants ≥35 weeks, suggesting that admission decisions are influenced not only by neonatal illness but also by local practice patterns [43]. Yet, the threshold of 35 weeks gestation indicates NICU admissions of clinically significant neonatal morbidity rather than admission based on prematurity only. In a recent cohort of late-preterm infants, 63% of infants born at 35 weeks and 78% of those born at 36 weeks remained in well-newborn care throughout hospitalization, whereas infants born at 34 weeks were managed in higher-acuity settings in significantly higher proportions [22]. Thus, the finding that in the novel classification, there was no prominent difference in mean gestational age at delivery between severe and mild abruption can be explained by the inherent properties of our classification. Nevertheless, this did not affect the severity of the cases identified by our classification.

Notably, perinatal death was the major contributor for the definition of severe cases. Previous literature suggests that stillbirth is a common catastrophic outcome of placental abruption, with reported rates ranging from 3.4% to 51.8% [12]. Furthermore, an increase in the extension of placental separation was associated with higher risk of stillbirth, suggesting that fetal demise reflects the severity of the underlying disease process [42].

Collectively, we identified only approximately 30% of placental abruption cases as severe. The group of severe abruptions based on our classification had a higher rate of severe and mild preeclampsia cases and lower mean concentrations of maternal fibrinogen and platelet counts in comparison to that based on Anath et al.’s classification. Thus, we have set a higher but comprehensive threshold for the diagnosis of severe abruption, generating a clear distinction of the severe cases facilitating the implementation of the novel classification in future studies and clinical settings.

Importantly, reliable data are essential for counseling women with placental abruption about recurrence risk, yet uncertainty remains. A prior abruption is the strongest predictor of recurrence; race and smoking have also been implicated, whereas hypertensive disorders increase overall risk but not recurrence [14]. Our findings demonstrate for the first time that severe abruption in the index pregnancy is an independent risk factor for recurrence and is consistent with previous reports regarding hypertensive disorders [14]. As a predictor of recurrence, our classification appears to more effectively distinguish patients with a greater underlying pathological burden from those with less severe forms of placental abruption.

### 4.3. Clinical Implications

The classification we developed performs well in identifying a subgroup of patients with placental abruption who exhibit severe features, with a higher proportion of epidemiological, clinical, and laboratory features associated with adverse outcomes. Hence, it may aid clinicians in identifying patients at a higher risk of postpartum complications, facilitating the need for intensive monitoring, interventions, or transfer to higher levels of care. It is important to note that although different cohorts with a higher prevalence of placental abruption, such as those in the USA, may result in a different proportion of severe disease [1,2], these differences can be mitigated since our classification is based on global severe obstetric features supporting its broader applicability [19,20,21,24,25,26].

Moreover, our findings suggest that the application of this classification may help identify women at an increased risk of recurrent placental abruption in subsequent pregnancies, allowing for more precise risk stratification and tailored follow-up.

### 4.4. Research Implications

Following its validation by others, the presented classification has the potential to generate the needed common terminology for reporting on placental abruption severity and outcomes, thereby facilitating epidemiological research through standardized severity classification and improving the comparability of findings across populations and studies. In addition, the differentiation of mild from severe forms of abruption, based on large-scale laboratory and clinical findings, may provide insights into the pathophysiology of placental abruption, fostering research on preventative measures or therapeutic targets.

However, as this classification is based on outcome data, further research is required to develop an antepartum classification model that predicts these outcomes and enables early intervention when needed.

### 4.5. Strengths and Limitations of the Study

The major limitation of our study is its retrospective nature and the long-time span of dataset collection, which precluded us from incorporating all available data points due to incomplete datasets. However, the large-scale, data-based classification remains clinically relevant, simple, and effective in differentiating subtypes of placental abruption. It also provides new evidence regarding the objective evaluation of placental abruption.

Another limitation is that it includes only outcome data and, therefore, is not yet applicable in the antenatal setting. Nonetheless, it is easy to implement in future studies as it relies on a small number of simple criteria, which may help address differences between centers globally. While additional research is warranted, it is worth noting that our medical center serves as a significant tertiary obstetric center, with approximately 17,000 deliveries per year from diverse populations.

## 5. Conclusions

We present a novel and clinically relevant placental abruption severity classification that is easy to use and implement. Moreover, severe abruption by our classification is an independent risk factor for recurrence abruption in subsequent pregnancies.

## Figures and Tables

**Table 1:** Maternal Demographic Characteristic and Medical History By Placental Abruption Classification.

		Ananth et al. Classification		Novel Classification	
**Characteristic**	Comparison Group, N = 296,714 ^1^	Mild Abruption, N = 279 ^1^	Severe Abruption, N = 1288 ^1^	Mild Abruption, N = 1118 ^1^	Severe Abruption, N = 449 ^1^
Age (years)	29.0 ± 5.8	31.5 ± 5.7 ^a,c,d^	30.0 ± 6.2 ^b,c,d^	30.3 ± 6.1 ^a,d^	30.1 ± 6.1 ^b,d^
Gravidity	3 (2,5)	4 (2,6.8) ^a,d^	4 (2,6) ^b,d^	3 (2,6) ^a,c,d^	4 (2,7) ^b,c,d^
Parity	3 (2,4)	3. (2,5) ^a,d^	3 (2,5) ^b,d^	3 (2,5) ^a,c,d^	4 (2,6) ^b,c,d^
Previous Cesarean section	14% (40,776)	25% (71)^a,d^	24% (308) ^b^	23% (262) ^a,d^	26% (117) ^b,d^
History of perinatal death	4.4% (12,983)	10% (29) ^a,d^	10% (130) ^b,d^	8.7% (97) ^a,c,d^	14% (62) ^b,c,d^
Recurrent Pregnancy Loss	3.6% (10,665)	5.4% (15) ^d^	6.8% (88) ^b,d^	6% (67) ^a,d^	8% (36) ^b,d^
History of Placental Abruption	0.3% (901)	3.6% (10) ^a,d^	2.1% (27) ^b,d^	1.9% (21) ^a,d^	3.6% (16) ^b,d^
Infertility Treatment	2.0% (5795)	3.9% (11) ^a,d^	3.5% (45) ^b,d^	4.4% (49) ^a,c,d^	1.6% (7) ^c,d^
Chronic Hypertension	0.9% (2660)	1.1% (5) ^a,d^	2.3% (30) ^b,d^	2.2% (25) ^a,d^	1.8% (8) ^d^

^1^ Mean ± (SD); Median (IQR) % (n), ^a^ significant difference between comparison group and mild abruption, ^b^ significant difference between comparison group and severe abruption, ^c^ significant difference between severe abruption and mild abruption, ^d^ Comparison of all three groups.

**Table 2:** Pregnancy And Delivery Characteristics according to placental abruption classification.

		Ananth et al. Classification		Novel Classification	
Characteristic	Comparison Group, N = 296,714 ^1^	Mild Abruption, N = 279 ^1^	Severe Abruption, N = 1288 ^1^	Mild Abruption, N = 1118 ^1^	Severe Abruption, N = 449 ^1^
2nd Trimester Bleeding	0.7% (2064)	1.8% (5) ^c,d^	17% (221) ^b,c,d^	14% (161) ^a,d^	14% (65) ^b,d^
Gestational Diabetes Miletus A	3.3% (9865)	5% (14)	3.8% (49)	4.4% (49)	3.1% (14)
Oligohydramnios	2.4% (7034)	2.5% (7) ^d^	5.5% (70) ^b,d^	5.1% (57) ^a,d^	4.7% (21) ^b,d^
Hydramnios	1.9% (5645)	5.4% (15) ^a,d^	3.3% (43) ^b,d^	3.8% (43) ^a,d^	3.3% (15) ^b,d^
Preterm-PROM	11% (33,371)	11% (32)	12% (159)	14% (157) ^a,c,d^	7.6% (34) ^b,c,d^
Mild Preeclampsia	2.9% (8585)	3.6% (10)	4.3% (56) ^b^	3.4% (38) ^c,d^	6.2% (28) ^b,c,d^
Severe Preeclampsia	0.9% (2798)	2.5% (7) ^a,c,d^	9.4% (121) ^b,c,d^	6.5% (73) ^a,c,d^	12% (55) ^b,c,d^
Multi-Fetal Gestation	2.0% (5897)	2.6% (8)	2.8% (35)	3.0% (34) ^a,d^	2.0% (9) ^d^

^1^ Mean± (SD); % (n), ^a^ significant difference between comparison group and mild abruption, ^b^ significant difference between comparison group and severe abruption, ^c^ significant difference between severe abruption and mild abruption, ^d^ Comparison of all three groups. Abbreviation: PROM—Premature Rupture of Membranes.

**Table 3:** Neonatal characteristics according to placental abruption classifications.

		Ananth et al. Classification		Novel Classification	
Characteristic	Comparison Group, N = 302,708 ^1^	Mild Abruption, N = 286 ^1^	Severe Abruption, N = 1327 ^1^	Mild Abruption, N = 1156 ^1^	Severe Abruption, N = 457 ^1^
Gestational Age at Delivery (Weeks)	38.9 ± 2.2	38.5 ± 2.2 ^a,c,d^	33.5 ± 4.8 ^b,c,d^	34.3 ± 5 ^a,c,d^	34.9 ± 4.9 ^b,c,d^
Apgar score 1 min < 5	2.7% (8200)	19% (53) ^a,c,d^	31% (408) ^b,c,d^	26% (299) ^a,c,d^	35% (162) ^b,c,d^
Apgar score 5 min < 7	1.2% (3746)	7.3% (21) ^a,c,d^	19% (246) ^b,c,d^	12% (141) ^a,c,d^	28% (126) ^b,c,d^
Birth Weight(gr)	3156.4 ± 560.3	3081.6 ± 533 ^a,c,d^	2018.9 ± 880 ^b,c,d^	2261.4 ± 920 ^a,d^	2071.1 ± 920 ^b,c^
LGA	6.2% (18,886)	7.7% (22) ^c,d^	2.9% (38) ^b,c,d^	4.1% (47) ^a,d^	2.8% (13) ^b,d^
SGA	3.5% (10,707)	0% (0) ^a,c,d^	12% (164) ^a,b,d^	9.2% (106) ^a,c,d^	13% (58) ^b,c,d^
Non-Vertex Presentation	3.2% (9644)	5.2% (15) ^a,d^	9.3% (124) ^b,d^	8% (93) ^a,d^	10% (46) ^b,d^

^1^ Mean± (SD); % (n), ^a^ significant difference between comparison group and mild abruption, ^b^ significant difference between comparison group and severe abruption, ^c^ significant difference between severe abruption and mild abruption, ^d^ comparison of all 3 groups. Abbreviations: SGA—Small for Gestational Age, LGA—Large for Gestational Age. N represents newborns and not women in the table.

**Table 4:** Laboratory Parameters By Placental Abruption Classification.

		Ananth et al. Classification		Novel Classification	
Characteristic	Comparison Group, N = 296,714 ^1^	Mild Abruption, N = 279 ^1^	Severe Abruption, N = 1288 ^1^	Mild Abruption, N = 1118 ^1^	Severe Abruption, N = 449 ^1^
Hemoglobin (mg/dL)	11.8 ± 1.4	11.8 ± 1.7 ^c,d^	11.3 ± 1.5 ^b,c,d^	11.5 ± 1.4 ^a,c,d^	11 ± 1.6 ^b,c,d^
Hematocrit (%)	35.8 ± 3.5	35.5 ± 3.6 ^c,d^	34 ±4.1 ^b,c,d^	34.7 ± 3.8 ^a,c,d^	33.2 ± 4.6 ^b,c,d^
Platelets (10^3^/uL)	232.5 ± 68.4	223.4 ± 62 ^d^	227 ± 68.3 ^b,d^	232.1 ± 64.6 ^c,d^	209.8 ± 72.0 ^b,c,d^
Thrombocytopenia < 100 × 10^3^/uL	0.5% (1454)	0% (0) ^c,d^	1.9% (25) ^b,c,d^	0.5% (6) ^c,d^	4.2% (19) ^b,c,d^
Fibrinogen (mg/dL)	594.2 ± 120.9	558.4 ± 107 ^a,c,d^	502.3 ± 156 ^b,c,d^	547.5 ± 125 ^c,d^	431.7 ± 170 ^b,c,d^
Hypofibrinogenemia < 100 mg/dL	<0.1% (3)	0% (0) ^d^	0.4% (5) ^b,d^	0% (0) ^a,c,d^	2.0% (5) ^b,c,d^

^1^ Mean ± (SD); % (n), ^a^ significant difference between comparison group and mild abruption, ^b^ significant difference between comparison group and severe abruption, ^c^ significant difference between severe abruption and mild abruption, ^d^ comparison of all three groups.

**Table 5:** Current and Novel Severity Criteria By Placental Abruption Severity.

		Ananth et al. Classification			Novel Classification		
	Characteristic	Mild Abruption, N = 279 ^1^	Severe Abruption, N = 1288 ^1^	*p*-Value	Mild Abruption, N = 1118 ^1^	Severe Abruption, N = 449 ^1^	*p*-Value
	Blood Transfusion ≥ 4 Units	0% (0)	3.3% (43)	0.004	0% (0)	9.6% (43)	<0.001
	Intensive Care Unit Admission	0.4% (1)	3.3% (42)	0.004	0% (0)	9.6% (43)	<0.001
	Birth Asphyxia	2.2% (6)	2.7% (35)	0.7	0% (0)	9.1% (41)	<0.001
	Umbilical Cord Blood pH < 7.0	4.3% (12)	5.4% (70)	0.4	0% (0)	20% (82)	<0.001
	Neonatal Intensive Care Unit Admission After 35 Weeks	6.5% (18)	7.5% (96)	0.8	0% (0)	25.4% (114)	<0.001
	Perinatal Death	0% (0)	20% (264)	<0.001	0% (0)	59% (264)	<0.001
	DIC	0% (0)	6.1% (79)	<0.001	0% (0)	18% (79)	<0.001
	Hysterectomy	0% (0)	1% (13)	0.09	0% (0)	2.9% (13)	<0.001
	Maternal Death	0% (0)	<0.1% (1)	>0.9	0% (0)	0.2% (1)	0.3
	Any Blood Transfusion	0% (0)	23% (293)	<0.001	10% (117)	39% (176)	<0.001
	Hypovolemic Shock	0% (0)	0.7% (9)	0.3	<0.1% (1)	1.8% (8)	<0.001
	Renal Failure	0% (0)	0.7% (9)	0.2	0% (0)	2% (9)	<0.001
	Non-reassuring Fetal Status	0% (0)	34% (442)	<0.001	30% (333)	24% (109)	0.03
	FGR	0% (0)	8.3% (107)	<0.001	6.9% (77)	6.7% (30)	0.9
	Preterm Delivery	0% (0)	66% (854)	<0.001	53% (594)	58% (260)	0.09
	SGA	0% (0)	12% (159)	<0.001	9.1% (101)	13% (58)	0.02

^1^ % (n), Abbreviations: FGR—Fetal growth restriction, SGA—Small for Gestational Age. 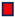
—Exclusive for the novel classification. 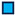
—Exclusive for the Ananth et al. classification. 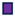
—Overlap between the classifications.

**Table 6:** Multivariable Logistic Regression Analysis for Prediction of Recurrent Placental Abruption According to The Novel and Ananth’s classifications.

	Novel Classification			Ananth et al. Classification		
Variable	OR	Confidence Interval (95%)	*p*-Value	OR	Confidence Interval (95%)	*p*-Value
Severe Abruption	1.82	1.05–3.14	0.03	1.602	0.716–3.582	0.25
Parity	1.01	0.92–1.11	0.77	1.026	0.936–1.125	0.58
Chronic Hypertension	0.85	0.11–6.42	0.87	0.774	0.102–5.854	0.8
Preeclampsia	0.74	0.31–1.78	0.51	0.801	0.337–1.905	0.62

## Data Availability

The data presented in this study are not publicly available due to privacy and ethical restrictions.
